# Ca_V_1.3 L-Type Calcium Channels Increase the Vulnerability of Substantia Nigra Dopaminergic Neurons in MPTP Mouse Model of Parkinson’s Disease

**DOI:** 10.3389/fnagi.2019.00382

**Published:** 2020-01-17

**Authors:** Aditi Verma, Vijayalakshmi Ravindranath

**Affiliations:** ^1^Centre for Neuroscience, Division of Biological Sciences, Indian Institute of Science, Bangalore, India; ^2^Centre for Brain Research, Indian Institute of Science, Bangalore, India

**Keywords:** neurodegeneration, SNpc, MPTP, Ca_V_1.3_42A_, gene expression, alternative splicing

## Abstract

Mechanisms underlying the selective vulnerability of dopaminergic (DA) neurons in the substantia nigra pars compacta (SNpc) over those in the ventral tegmental area (VTA) to degeneration in Parkinson’s disease (PD) remain poorly understood. DA neurons of SNpc and VTA are autonomous pacemakers but pacemaking in SNpc but not in VTA is accompanied by calcium influx through L-type calcium channel, Ca_V_1.3 contributing to increased intracellular calcium and hence to cell death. Ca_V_1.3_42A_, an alternatively spliced short variant of Ca_V_1.3 has increased calcium influx. We, therefore studied the role of Ca_V_1.3_42_ (full-length channel) and Ca_V_1.3_42A_ in mouse SNpc in PD pathogenesis by quantifying mRNA levels of Ca_V_1.3_42_ and Ca_V_1.3_42A_ in SNpc and followed the change in their levels in MPTP induced parkinsonism mouse model. Using *in situ* hybridization and immunohistochemistry we observed the localization of mRNA of Ca_V_1.3_42_ and Ca_V_1.3_42A_ in tyrosine hydroxylase (TH) positive DA neurons. Further, mRNA levels of Ca_V_1.3_42A_ were higher in SNpc as compared to the cortex. Upon MPTP treatment, mRNA levels of Ca_V_1.3_42_ and Ca_V_1.3_42A_ maintained their levels in SNpc in spite of the loss of ~50% of the DA neurons. This indicates that the expression of Ca_V_1.3_42_ and Ca_V_1.3_42A_ is maintained at a robust level during the degenerative process in the parkinsonism model.

## Introduction

Parkinson’s Disease (PD) is a debilitating movement disorder characterized by locomotor deficits including resting tremor, bradykinesia, rigidity and postural instability. Motor deficits result from the loss of dopaminergic (DA) neurons in the large midbrain nucleus, substantia nigra pars compacta (SNpc). SNpc DA neurons degenerate while neighboring DA neurons from the ventral tegmental area (VTA) remain relatively protected from neurodegeneration (Hirsch et al., 1988; Gibb and Lees, 1991; Dauer and Przedborski, 2003; Brichta and Greengard, 2014). A number of mechanisms have been implicated in PD neurodegeneration, namely oxidative stress possibly in relation with iron deposition (Sofic et al., 1988; Ayton and Lei, 2014), abnormal dopamine metabolism (Michel and Hefti, 1990; Pifl et al., 2014), mitochondrial dysfunction (Schapira et al., 1990; Park et al., 2009; Michel et al., 2016) and disruption of proteasomal or autophagic catabolism (Sherman and Goldberg, 2001; Betarbet et al., 2005; Carvalho et al., 2013; Michel et al., 2016). However, in PD none of these mechanisms provides in itself an explanation for the greater vulnerability of SNpc dopaminergic neurons over DA neurons in VTA.

There is also evidence to suggest that dysregulation in calcium homeostasis could potentially account for this selectivity (Surmeier et al., 2010, 2011, 2017; Schapira, 2013; Surmeier and Schumacker, 2013). In fact, it has been reported that DA neurons from VTA express higher levels of the calcium buffering protein, calbindin-D28K (Damier et al., 1999). Besides, DA neurons in SNpc but not in VTA are characterized by pacemaking activity that is accompanied with activity of a subset of L-type voltage-dependent calcium channels having a Ca_V_1.3 pore that elevates intracellular Ca^2+^ (Nedergaard and Greenfield, 1992; Kang and Kitai, 1993; Mercuri et al., 1994; Chan et al., 2007; Surmeier et al., 2017). However, pacemaking in VTA DA neurons appears to depend primarily on HCN/voltage-gated sodium channels (Khaliq and Bean, 2010) and the cytosolic Ca^2+^ in VTA DA neurons during pacemaking is reported to be significantly less as compared to that in DA neurons in SNpc (Guzman et al., 2018). This has led to the hypothesis that Ca^2+^-dependent pacemaking along with a poor calcium buffering capacity may cause calcium overload through L-type Ca_V_1.3 channel activation in most vulnerable DA SNpc neurons making them preferentially at risk to degeneration (Surmeier et al., 2010, 2017; Liss and Striessnig, 2019).

Several alternatively spliced variants of the full-length channel Ca_V_1.3_42_ have been reported, among them is Ca_V_1.3_42A_, a short splice variant that incorporates in a mutually exclusive manner, exon 42A instead of exon 42 (Bock et al., 2011; Huang et al., 2013). Exon 42A contains a stop codon and translation of the protein terminates before encoding the C-terminal modulatory domain, which results in a splice variant product with different electrophysiological properties compared to the full-length channel. The calcium current density through Ca_V_1.3_42A_ is about 2.5 times greater than in Ca_V_1.3_42_ (Singh et al., 2008). Further, it has been reported that the activation range of Ca_V_1.3_42A_ is shifted to a more negative potential by ~10 mV (Singh et al., 2008). Moreover, these variants show greater insensitivity to inhibition by dihydropyridines (DHPs) as compared to other C-terminus variants and the full-length channel (Huang et al., 2013). Thus, the activation potential of Ca_V_1.3_42A_ is closer to the resting membrane potential and there is greater calcium influx through this variant as compared to other C-terminus variants and full-length channel (Tan et al., 2011; Huang et al., 2013). Further, the presence of these channels has been demonstrated in the mouse and human brain (Singh et al., 2008; Bock et al., 2011).

Owing to the atypical electrophysiological properties of Ca_V_1.3_42A_, we hypothesized that the expression of this splice variant in SNpc could potentially play a role in DA cell death by causing perturbations in calcium homeostasis. To this aim we studied the expression of mRNA encoding Ca_V_1.3_42A_ and Ca_V_1.3_42_ (full-length) in midbrain DA neurons in naïve mice and evaluated if the transcription of these channels was affected during neurodegeneration mediated by MPTP in mouse model of PD.

## Materials and Methods

### Animals and MPTP Dosing

Animal experiments were carried out on C57BL/6J male mice (3–4 months; 25–30 g) procured from Central Animal Facility of Indian Institute of Science, Bangalore, India. All animal experiments were carried out according to institutional guidelines for the use and care of animals. Animal experiments were approved by the institutional animal ethical review board, named “Institutional Animal and Ethics Committee” of the Indian Institute of Science (Protocol# CAF/Ethics/267/2012). Handling of animals was done according to the guidelines of the Committee for the Purpose of Control and Supervision of Experiments on Animals (CPCSEA), Government of India. All experiments were performed in adherence to ARRIVE guidelines. All efforts were made to minimize animal suffering, reduce the number of animals used and to use alternatives to *in vivo* techniques, if available. Animals were housed in groups and had access to pelleted diet and water, *ad libitum*. The sample size for experiments with untreated animals was 6–11. For the MPTP mouse model of dopaminergic loss (Jackson-Lewis and Przedborski, 2007), 30 mg/kg body weight 1-methyl-4-phenyl-1,2,3,6-tetrahydropyridine (MPTP; Sigma–Aldrich Cat# M0896) dissolved in normal saline was given subcutaneously to the mice as a single dose or daily for 14 days. MPTP injections were carried out in an isolated clean air room in the Central Animal Facility, Indian Institute of Science, Bangalore, India. The controls were injected with an equivalent volume of normal saline. Animals were allocated to treated or control groups in a random manner. The sample sizes for MPTP experiments were 4–11 for each group. Animals were sacrificed 24 h after the last MPTP dose.

Adequate safety precautions were followed in the proper handling of MPTP during preparation and injection, and in the disposal of materials and samples contaminated with MPTP and its metabolites. Protective gear was worn during the preparation and injection of MPTP. Syringes and needles that were used for injection were incinerated after a single-use. Mice undergoing MPTP treatment were housed in separate cages and the contaminated bedding material and feed were incinerated upon disposal.

### Mouse Brain Dissection

Animals were decapitated following cervical dislocation. Cortex, ventral midbrain and striatum were dissected out under cold and sterile conditions. For dissection of SNpc, the whole mouse brain was placed on mouse brain matrix (Ted Pella, Inc., Cat# 15050) and 1 mm thick slices of the brain were obtained. Then, SNpc was dissected out from these slices under a dissecting stereomicroscope using anatomical markers.

### RNA Isolation and cDNA Synthesis

All the reagents and glassware used for RNA isolation, cDNA synthesis, *in situ* hybridization and immunohistochemistry were made RNAse free. RNA from mouse brain tissue was isolated using TRIzol reagent (Invitrogen Cat# 15596018) and bromochlorophenol (BCP; Moelcular Research Centre, Inc., Cat# BP151; Chomczynski and Sacchi, 2006). Total RNA (500 ng) was used for first-strand cDNA synthesis using random hexamers, dNTPs and reverse transcriptase from the High capacity cDNA reverse transcription kit (Applied Biosystems Cat# 4368814).

### Quantitative Real-Time PCR

Quantitative real-time PCR (qRT-PCR) was performed using SYBR green chemistry with primer pairs designed to distinguish the full-length Ca_V_1.3 and splice variant. The nucleotide sequences for primers used for mouse gene expression analysis and the PCR conditions are provided in Supplementary Tables S1, S2, respectively. Further, the specificity of the primers as assessed by the presence of a single band at the desired size measured through gel electrophoresis has been represented in Supplementary Figure S1. Three endogenous controls, namely 18S rRNA, β-actin and GAPDH were used for normalization when cDNA from untreated mouse tissue was analyzed. β-actin and/or GAPDH normalization was performed in subsequent experiments as reported. Further, cell-type-specific normalizations were performed with tyrosine hydroxylase (TH), DAT, GAD1, and VGlut2. The samples were analyzed in duplicates or triplicates. Data from all samples have been reported and no exclusion of outliers has been performed.

### Fluorescent *in situ* Hybridization (FISH) and Immunohistochemistry

Male C57BL/6J mice brains were isolated and fixed in 4% paraformaldehyde (w/v) for 12 h following decapitation after cervical dislocation. Fixed brains were then allowed to sink in 30% sucrose before embedding in tissue freezing system (Leica Microsystems Nussloch GmbH Cat# 0201 08926). Coronal sections measuring 14 μm in thickness were cut through midbrain under RNAse free conditions using a Cryostat (Leica Microsystems). The sections were hydrated, acetylated and treated with 25 μg of proteinase K (Roche Cat# 03115852001) for 7 min at 37°C. The sections were then rinsed with phosphate buffer and dehydrated using ethanol gradient. Digoxigenin-labeled sense (control) and antisense RNA probes were synthesized using SP6 and T7 polymerases (Roche Cat# 11175025910), respectively from Ca_V_1.3_42_ and Ca_V_1.3_42A_ cDNA sequences that were cloned into dual promoter pCRII vector (Invitrogen Cat# K206001). The sequences of the primers used for Ca_V_1.3_42_ and Ca_V_1.3_42A_ amplification are as follows: mouse Ca_V_1.3_42_, full-length (NM_028981.2; Forward, GGGAAAGTACCCTGCGAAGAACACC; Reverse, GGATTTCTGGCCCAATGTCATGCAG) and Ca_V_1.3_42A_, splice variant (Forward, CAGATGCTTGAACGGATGCTTTAG; Reverse, CTTCCTTCCGGAGGAGTGC). The sections were hybridized with sense and antisense probes (100 ng/μl) overnight in a humid chamber at 45°C followed by washing, incubation with 0.5% blocking agent (from Invitrogen TSA Kit #21 Cat# T20931). Signal was developed using a peroxidase-labeled anti-DIG antibody (Roche Cat# 11207733910) at a concentration of 1 in 250 followed by tyramide signal amplification (Invitrogen TSA Kit #21 Cat# T20931) and finally incubation with fluorescein-conjugated streptavidin (Vector Laboratories Cat# SA-5001) at a concentration of 1 in 500. Absence of fluorescence signal on the sections hybridized with the sense probes has been represented in Supplementary Figure S2.

Immunohistochemistry (IHC) was performed for investigating the co-localization of the expression of calcium channel isoforms with marker of DA neurons, TH. IHC was performed on the same sections on which FISH was performed. The sections were first rinsed in phosphate buffer followed by blocking and overnight incubation in anti-TH rabbit antibody (Millipore Cat# AB152, RRID:AB_390204). Sections were then washed and incubated in Goat Anti-Rabbit IgG H+L (Alexa Fluor^®^ 594; Thermo Fisher Scientific, Cat# A-21207, RRID:AB_141637) followed by washing. The sections were then mounted in Vectashield^®^ mounting medium (Vector Laboratories Cat# H-1000) and imaged as z-stacks using a Zeiss LSM 780 confocal microscope using LD LCI plan-apochromat 25×/0.8 Oil objective using 488 nm and 594 nm lasers for low magnification images and plan-apochromat 100×/1.4 Oil objective using 488 nm and 594 nm lasers for high magnification images. The maximum intensity projection that is used for representation was derived using Zeiss ZEN black software and orthogonal reconstruction was performed using Zeiss ZEN blue software.

### Statistical Analyses

Analysis of relative gene expression from the qRT-PCR data was done using ΔΔCt method. The thresholds were set manually. Data were analyzed using Graphpad Prism (Graphpad Prism Inc., San Diego, CA, USA). Shapiro–Wilk test was performed on all datasets to test for normality and statistical tests were carried out accordingly. Statistical significance was determined using one-way ANOVA followed by student’s Newman–Keuls post-test for multiple comparisons and student’s *t-*test for comparison between two groups for data that passed normality test. Two-tailed Mann–Whitney-U test was used for comparison between two groups when the data did not pass the test for normality.

## Results

### Ca_V_1.3_42A_ and Ca_V_1.3_42_ Expression in Ventral Midbrain, Cortex, and Striatum in Mice

**Figure 1:**
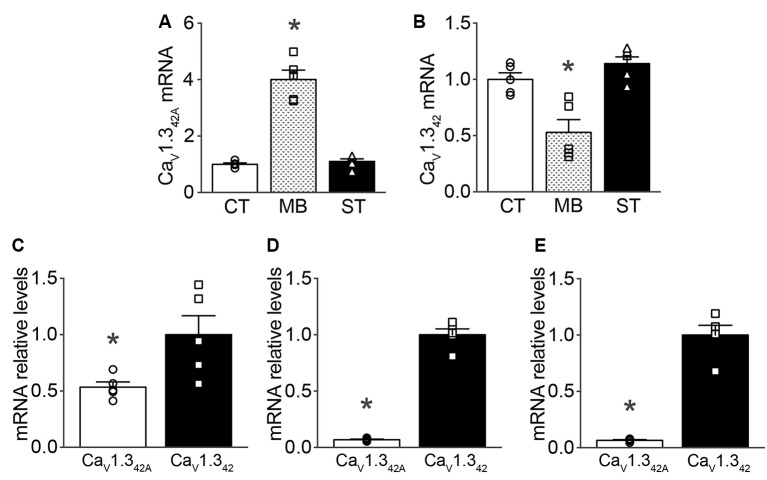
Ca_V_1.3_42_ and Ca_V_1.3_42A_ mRNA expression levels in mouse brain regions as measured by qRT-PCR.** (A)** Expression of Ca_V_1.3_42A_ (*F*_(2,12)_ = 15.54, *p* = 0.0005), **(B)** Ca_V_1.3_42_ (*F*_(2,12)_ = 73.22, *p* < 0.0001) in the ventral midbrain (MB) in comparison to expression profiles in the cortex (CT) and striatum (ST). Panels **(C–E)** describe the relative expression of Ca_V_1.3_42A_ to Ca_V_1.3_42_ in ventral midbrain (*p* = 0.0281, *t* = 2.675, *df* = 8), cortex (*p* < 0.0001, *t* = 17.78, *df* = 8) and striatum (*p* < 0.0001, *t* = 10.86, *df* = 8), respectively. Data were normalized to mean of 18S rRNA, β-actin and GAPDH as endogenous controls. Each dot in the figure represents an individual animal. One-way ANOVA with Newman–Keuls test was performed for multiple comparisons and unpaired, two-tailed student’s *t*-test was performed for pair-wise comparisons on the data. Data represented as mean ± SEM. *p < 0.05.

mRNA levels of Ca_V_1.3_42A_ and Ca_V_1.3_42_ were assayed in the ventral midbrain of C57BL/6J naïve mice using qRT-PCR. mRNA levels for Ca_V_1.3_42A_, the short splice variant of Ca_V_1.3 containing exon 42A, were four times greater in the ventral midbrain as compared to the cortex and striatum (Figure 1A). Conversely, mRNA levels of full-length Ca_V_1.3 (Ca_V_1.3_42_) were greater in the cortex and striatum than in the ventral midbrain (Figure 1B). When comparing relative mRNA levels for Ca_V_1.3_42A_ and Ca_V_1.3_42_, we found that Ca_V_1.3_42A_ mRNA levels represented about 0.5 times that of Ca_V_1.3_42_ in the ventral midbrain (Figure 1C). However, in the cortex and striatum mRNAs encoding Ca_V_1.3_42A_ were much less abundant (approximately 0.06 times) than mRNAs for Ca_V_1.3_42_ (Figures 1D,E, respectively).

### Co-localization of Ca_V_1.3_42A_ and Ca_V_1.3_42_ With TH Positive Dopaminergic Neurons in SNpc

**Figure 2:**
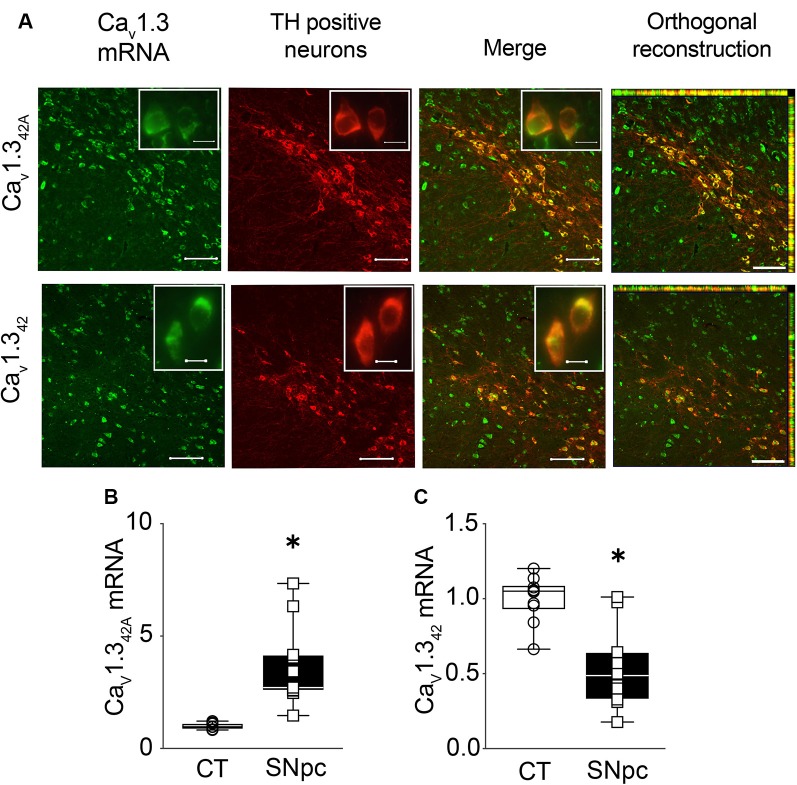
Expression of Ca_V_1.3_42A_ and Ca_V_1.3_42_ in the substantia nigra pars compacta (SNpc) from control mouse brain.** (A)** Ca_V_1.3_42A_ and full-length L-type calcium channel Ca_V_1.3_42_ mRNA detected within DA neurons expressing tyrosine hydroxylase (TH) in the SNpc using RNA fluorescence *in situ* hybridization and TH immunohistochemistry, respectively. Insets represent high-magnification images of the TH positive neurons. **(B)** mRNA levels for Ca_V_1.3_42A_ are significantly higher in the SNpc as compared to cortex (CT; Mann–Whitney-*U* = 0, *n*_1_ = 10, *n*_2_ = 11, *p* < 0.0001, two-tailed), **(C)** while mRNA levels for Ca_V_1.3_42_ are significantly lower in SNpc in comparison to CT (*p* < 0.0001, *t* = 4.941, *df* = 19). mRNA levels of calcium channels were normalized to mRNA levels of the house-keeping gene GAPDH. Each dot in the graphs represents an individual animal. Data represented as box-whiskers plots where each box represents quartiles with the line indicating median. Whiskers show the absolute range. *n* = 10–11. *p < 0.05. Scale bar = 100 μm, scale bar for inset = 10 μm.

The available antibodies against Ca_V_1.3 are highly non-specific. Therefore, *in situ* hybridization was used to identify and localize the expression of Ca_V_1.3_42A_ and Ca_V_1.3_42_ in the midbrain. Combined fluorescent *in situ* hybridization for Ca_V_1.3_42A_ and immunohistochemistry for TH allowed us to demonstrate that mRNAs encoding the short splice variant were present in SNpc DA neurons in control mice as indicated by orthogonal reconstruction (Figure 2A). Likewise, Ca_V_1.3_42_ mRNA was also co-localized with TH positive neurons in SNpc as seen from orthogonal reconstruction (Figure 2A). When using qRT-PCR, we found that mRNA levels of Ca_V_1.3_42A_ were approximately four-fold higher in the SNpc as compared to the cortex (Figure 2B). In contrast, mRNA levels for the full-length channel Ca_V_1.3_42_ were lower in the SNpc as compared to the cortex (Figure 2C). These observations are also consistent with mRNA expression data reported for Ca_V_1.3_42_ and Ca_V_1.3_42A_ in the midbrain and cortex in Figure 1.

### mRNA Levels of Ca_V_1.3_42A_ and Ca_V_1.3_42_ in SNpc After Acute Exposure to MPTP

**Figure 3:**
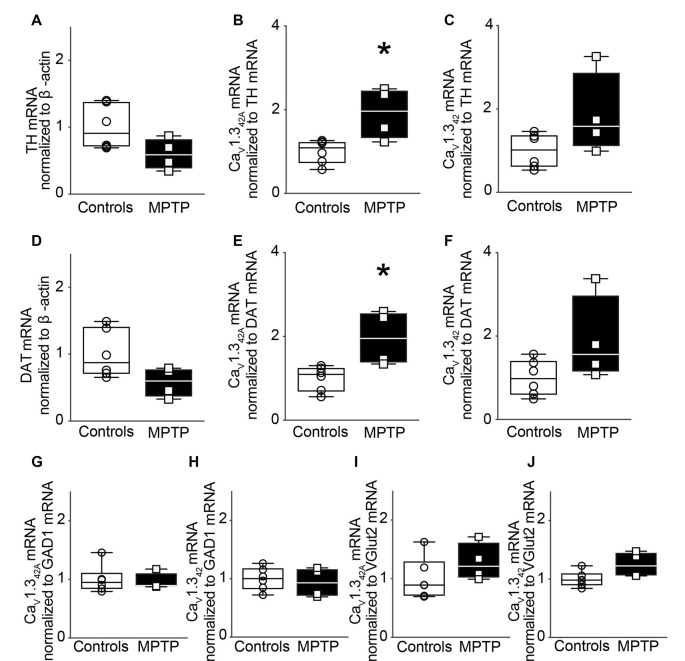
Modulation of Ca_V_1.3_42A_ but not Ca_V_1.3_42_ mRNA levels in the SNpc of MPTP-treated mice after 1 day of treatment.** (A)** TH mRNA expression in the SNpc of mice treated subcutaneously with a single dose of MPTP (30 mg/kg body weight) and sacrificed after 1 day. MPTP treatment did not lead to a change in TH mRNA levels in the SNpc (*p* = 0.0722, *t* = 2.070, *df* = 8). qRT-PCR data were normalized to mRNA levels of β-actin. **(B)** Relative mRNA levels for Ca_V_1.3_42A_ (*p* = 0.0121, *t* = 3.229, *df* = 8) increased in the SNpc in response to MPTP treatment and **(C)** Ca_V_1.3_42_ (*p* = 0.0901, *t* = 1.927, *df* = 8) did not change significantly when the mRNA signal was normalized to TH. **(D)** mRNA levels of DAT did not change upon MPTP treatment when normalized to mRNA levels of β-actin (*p* = 0.0733, *t* = 2.061, *df* = 8). **(E)** Relative mRNA levels for Ca_V_1.3_42A_ (*p* = 0.0126, *t* = 3.198, *df* = 8) increased in the SNpc in response to MPTP treatment while **(F)** Ca_V_1.3_42_ (*p* = 0.899, *t* = 1.929, *df* = 8) were not significantly different when the mRNA signal was normalized to DAT mRNA levels. **(G)** mRNA levels for Ca_V_1.3_42A_ (Mann–Whitney-*U* = 11, *n*_1_ = 6, *n*_2_ = 4, *p* = 0.9143, two-tailed) and **(H)** Ca_V_1.3_42_ (*p* = 0.6589, *t* = 0.4584, *df* = 8) were found to be unchanged in the SNpc of MPTP-treated mice upon normalization to GAD1. **(I)** mRNA levels for Ca_V_1.3_42A_ (*p* = 0.2405, *t* = 1.268, *df* = 8) and **(J)** Ca_V_1.3_42_ (*p* = 0.0562, *t* = 2.232, *df* = 8) were also found to be unchanged in the SNpc of MPTP-treated mice upon normalization to Vglut2. Each point represents an individual animal. For controls *n* = 6, for MPTP treated samples, *n* = 4. Unpaired, two-tailed student’s *t*-test or two-tailed Mann–Whitney-U test has been performed on each control vs. MPTP comparison. Data represented as box-whiskers plots where each box represents quartiles with the line indicating median. Whiskers show the absolute range. *p < 0.05.

MPTP, which is known to cause a selective loss of DA neurons in SNpc when administered to mice serves as a model of neurodegeneration akin to that seen in PD. In the present study, male C57BL/J6 mice received a single dose of MPTP (30 mg/kg body weight dose) administered subcutaneously. The animals were sacrificed 1 day after the dose. A single dose of MPTP did not result in the loss of TH mRNA levels in the SNpc (Figure 3A).

Subsequently, mRNA levels of Ca_V_1.3_42A_ and Ca_V_1.3_42_ were assessed in SNpc. However, normalization of Ca_V_1.3_42A_ and Ca_V_1.3_42_ mRNA to TH mRNA revealed that the expression of mRNA encoding Ca_V_1.3_42A_ was significantly increased in the SNpc (Figure 3B) while Ca_V_1.3_42_ presented no significant change in mRNA expression (Figure 3C).

These results were further validated by using dopamine transporter (DAT) as another marker for dopaminergic neurons. MPTP treatment did not result in significant loss of DAT mRNA transcript levels in SNpc (Figure 3D). Upon normalization of Ca_V_1.3_42A_ and Ca_V_1.3_42_ to DAT mRNA, there was a statistically significant increase in mRNA expression of Ca_V_1.3_42A_ (Figure 3E) but there was no significant difference in expression of Ca_V_1.3_42_ mRNA (Figure 3F). There was also no significant change when Ca_V_1.3_42A_ and Ca_V_1.3_42_ mRNA levels were normalized to mRNA levels for glutamate decarboxylase 1 (GAD1) and vesicular glutamate transporter 2 (Vglut2), markers of GABAergic (Figures 3G,H) and glutamatergic (Figures 3I,J) neurons, respectively. Our results indicate the differential expression of Ca_V_1.3_42A_ but not of Ca_V_1.3_42_ following acute exposure to MPTP.

### mRNA Levels of Ca_V_1.3_42A_ and Ca_V_1.3_42_ in SNpc After Sub-chronic Exposure to MPTP

**Figure 4:**
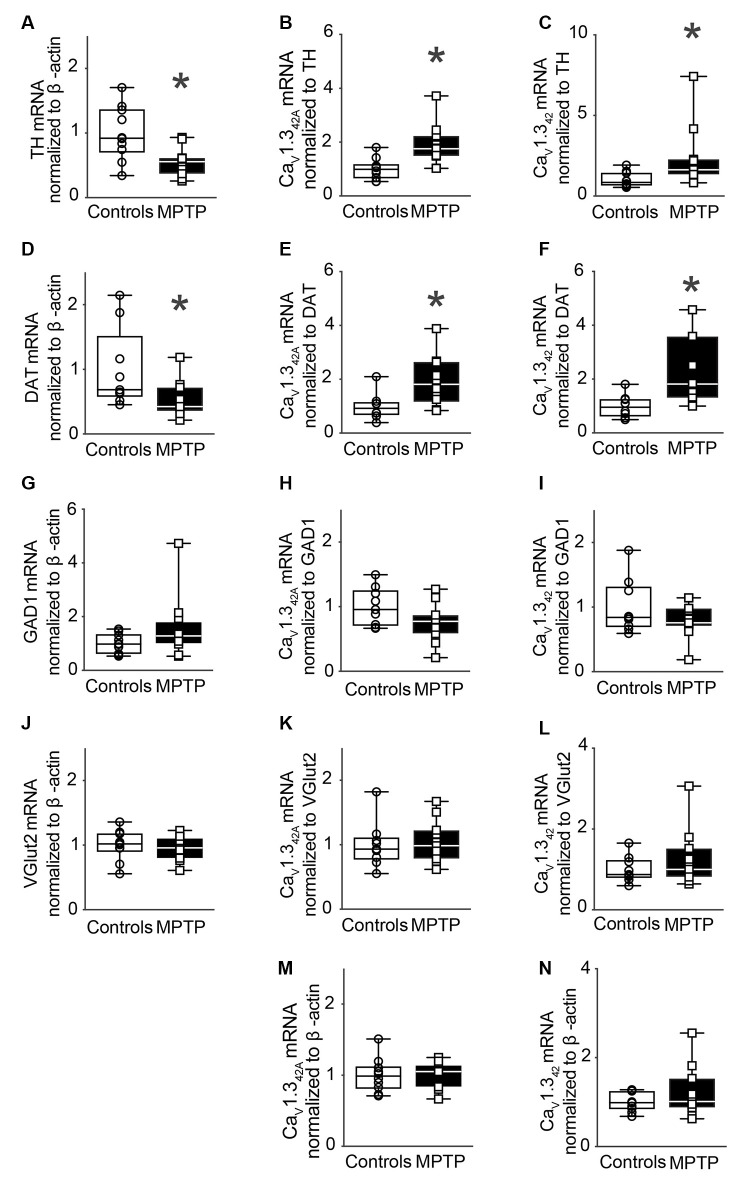
Modulation of Ca_V_1.3_42A_ and Ca_V_1.3_42_ mRNA levels in the SNpc of MPTP-treated mice after 14 days of treatment.** (A)** TH mRNA expression in the SNpc of mice treated subcutaneously with MPTP (30 mg/kg body weight) for 14 days. MPTP treatment led to a 45% reduction of TH mRNA levels in the SNpc (*p* = 0.0052, *t* = 3.152, *df* = 19). qRT-PCR data were normalized to mRNA levels of β-actin. **(B)** Relative mRNA levels for Ca_V_1.3_42A_ (*p* = 0.0048, *t* = 3.19, *df* = 19) and **(C)** Ca_V_1.3_42_ (Mann–Whitney *U* = 17, *n*_1_ = 10, *n*_2_ = 11, *p* = 0.0062, two-tailed) increased in the SNpc in response to MPTP treatment when the mRNA signal was normalized to TH. **(D)** mRNA levels of DAT were reduced by 47% upon MPTP treatment when normalized to mRNA levels of β-actin (Mann–Whitney-*U* = 19, *n*_1_ = 9, *n*_2_ = 11, *p* = 0.0200, two-tailed). **(E)** Relative mRNA levels for Ca_V_1.3_42A_ (*p* = 0.0227, *t* = 2.522, *df* = 16) and **(F)** Ca_V_1.3_42_ (*p* = 0.0114, *t* = 2.859, *df* = 16) increased in the SNpc in response to MPTP treatment when the mRNA signal was normalized to DAT mRNA levels. **(G)** mRNA levels of GAD1 remained unchanged when normalized to β-actin (Mann–Whitney-*U* = 41, *n*_1_ = 10, *n*_2_ = 11, *p* = 0.2276, two-tailed). **(H)** mRNA levels for Ca_V_1.3_42A_ (*p* = 0.0787, *t* = 1.864, *df* = 18) and **(I)** Ca_V_1.3_42_ (*p* = 0.2097, *t* = 1.301, *df* = 18) were found to be unchanged in the SNpc of MPTP-treated mice upon normalization to GAD1. **(J)** mRNA levels of VGlut2 remained unchanged when normalized to β-actin (*p* = 0.5594, *t* = 0.5936, *df* = 20). **(K)** mRNA levels for Ca_V_1.3_42A_ (*p* = 0.7443, *t* = 0.3312, *df* = 18) and **(L)** Ca_V_1.3_42_ (Mann–Whitney-*U* = 43, *n*_1_ = 9, *n*_2_ = 11, *p* = 0.6556, two-tailed) were found to be unchanged in the SNpc of MPTP-treated mice upon normalization to Vglut2. **(M)** mRNA levels for Ca_V_1.3_42A_ (*p* = 0.8383, *t* = 0.2069, *df* = 19) and **(N)** Ca_V_1.3_42_ (*p* = 0.2777, *t* = 1.118, *df* = 19) were found to be unchanged in response to MPTP treatment upon normalization to β-actin. Each point represents an individual animal. For controls *n* = 9–10, for MPTP treated samples, *n* = 10–12. Unpaired, two-tailed student’s *t*-test or two-tailed Mann–Whitney-U test was performed on each control vs. MPTP comparison. Data are represented as box-whiskers plots where each box represents quartiles with the line indicating median. Whiskers show the absolute range. *Indicates *p* < 0.05.

Further, to assess the effect of sub-chronic exposure to MPTP on the mRNA expression of Ca_V_1.3_42A_ and Ca_V_1.3_42_, male C57BL/J6 mice received MPTP that was administered subcutaneously once a day for 14 days. This treatment regimen resulted in a 45% loss of TH mRNA levels in the SNpc (Figure 4A).

mRNA levels of Ca_V_1.3_42A_ and Ca_V_1.3_42_ were then assessed in SNpc. Normalization of Ca_V_1.3_42A_ and Ca_V_1.3_42_ mRNA to TH mRNA resulted in significantly increased expression of mRNA encoding Ca_V_1.3_42A_ and Ca_V_1.3_42_, respectively, in the SNpc (Figures 4B,C). Further, MPTP treatment resulted in a 47% loss of DAT mRNA transcript levels in SNpc (Figure 4D). Upon normalization of Ca_V_1.3_42A_ and Ca_V_1.3_42_ to DAT mRNA, there was a statistically significant increase in mRNA expression (Figures 4E,F). Moreover, the increased expression of Ca_V_1.3_42A_ or Ca_V_1.3_42_ mRNA was not observed in the VTA from animals after sub-chronic MPTP exposure (Supplementary Figure S3). MPTP treatment did not change the expression of GAD1 (Figure 4G) and VGlut2 (Figure 4J). No significant difference in mRNA expression was observed when Ca_V_1.3_42A_ and Ca_V_1.3_42_ mRNA levels were normalized to mRNA levels for GAD1 (Figures 4H,I) and Vglut2 (Figures 4K,L) and β-actin (Figures 4M,N), respectively.

## Discussion

It has been postulated that Ca_V_1.3 L-type calcium channels, which contribute to the pacemaking activity of SNpc DA neurons may also play a role in their vulnerability to degeneration. Indeed, autonomous pacemaking leads to increase in basal mitochondrial oxidative stress in SNpc DA neurons, presumably as a direct consequence of the Ca^2+^ load (Foehring et al., 2009; Guzman et al., 2010; Surmeier et al., 2017; Liss and Striessnig, 2019). In support of this hypothesis, it has been shown that blocking Ca_V_1.3 with a calcium channel antagonist, isradipine, afforded protection against neurodegeneration in the MPTP, 6-OHDA and rotenone rodent model of PD (Chan et al., 2007, 2010; Ilijic et al., 2011). Further, Guzman et. al. have demonstrated that chronic, systemic isradipine treatment led to reduced cytosolic Ca^2+^ in SNpc DA neurons and lowered mitochondrial oxidant stress. It has been shown that knockdown of Ca_V_1.3 resulted in reduced dendritic Ca^2+^ oscillations in SNpc DA neurons elucidating their importance in the process (Guzman et al., 2018). This prompted us to study the expression of mRNAs encoding the full-length channel Ca_V_1.3_42_ and its C-terminally truncated splice variant Ca_V_1.3_42A_ in SNpc. Besides, we evaluated the transcripts of the two-channel isoforms in the MPTP mouse model of PD.

The qRT-PCR analysis revealed that Ca_V_1.3_42A_ transcripts were expressed more abundantly in the ventral midbrain including SNpc when compared to the cortex, whereas the opposite was true for Ca_V_1.3_42_ transcripts. Interestingly, mRNAs encoding the full-length channel Ca_V_1.3_42_ as well as its truncated splice variant Ca_V_1.3_42A_ were found to be selectively increased in the SNpc of MPTP-treated mice.

While the presence of the full-length isoform of Ca_V_1.3 channels Ca_V_1.3_42_ and that of its short splice variant Ca_V_1.3_42A_ has been reported in the mouse (Bock et al., 2011; Tan et al., 2011) and human brain (Singh et al., 2008), there is no study comparing the regional expression of the two mRNA isoforms in the brain. By coupling *in situ* hybridization to immunohistochemistry, we demonstrated that both Ca_V_1.3_42_ and Ca_V_1.3_42A_ mRNA expressed in TH positive neurons in the SNpc. Furthermore, we showed that mRNA levels for the splice variant Ca_V_1.3_42A_ were higher in mouse ventral midbrain as compared to cortex or striatum whereas the opposite was true for Ca_V_1.3_42_ transcripts. Ca_V_1.3_42A_ mRNA expression was also higher in the SNpc in comparison to the cortex in concurrence with the above. This indicates that the L-type calcium channel isoform Ca_V_1.3_42A_ substantially contributes to calcium influx in midbrain DA neurons in the SNpc. Since calcium current density through Ca_V_1.3_42A_ is about 2.5 times greater than the full-length channel isoform (Singh et al., 2008), one may assume that calcium influx through this channel is more pronounced in midbrain DA neurons than cortical neurons. It may be noted that the pacemaking activity inherent to DA neurons is accompanied by Ca_V_1.3 L-type calcium channels in the SNpc but not in the VTA where it relies on Na_V_/HCN channels (Guzman et al., 2010; Khaliq and Bean, 2010). This may render nigral DA neurons more vulnerable to calcium overload through activation of Ca_V_1.3_42A_ channels. The differential vulnerability between SNpc and VTA DA neurons may be further exacerbated by the fact that DA neurons from the VTA also contain high levels of the calcium buffering protein Calbindin-D28K while most vulnerable DA neurons in the SNpc are lacking this protein (Damier et al., 1999).

In the present study, we also examined mRNA expression changes, if any, of the L-type calcium channel and its variant in the SNpc in response to both acute and sub-chronic treatment regimens with the DA neurotoxin MPTP. In fact, the expression of Ca_V_1.3_42A_ was significantly higher in SNpc after 24 h of MPTP exposure although the levels of TH and DAT mRNA were not significantly affected (Figure 3). However, after 14 days of MPTP treatment, when the TH and DAT mRNA levels were reduced by approximately 50%, the mRNA levels of Ca_V_1.3_42_ and Ca_V_1.3_42A_ were maintained and were similar to vehicle controls. Since Ca_V_1.3_42_ and Ca_V_1.3_42A_ are predominantly expressed in SNpc by DA neurons, a ~50% reduction of the number of these neurons induced by sub-chronic MPTP treatment should be reflected by a significant decrease in transcripts encoding the two-channel isoforms. This was not the case, however, upon normalization to β-actin indicating that there is a compensatory increase in the expression of the channel transcripts in the surviving SNpc DA neurons. Further, when mRNA expression for Ca_V_1.3_42_ and Ca_V_1.3_42A_ was normalized to TH and DAT mRNA, we observed a significant increase in transcripts encoding Ca_V_1.3_42A_ after 1 day of single dose of MPTP and both channel isoforms after 14 days of exposure to MPTP. This suggested that DA neurons may produce more transcripts to possibly preserve their activity in surviving neurons in response to neurodegeneration triggers.

In rodents, while about 70% of SNpc neurons are DA neurons, about 29% of SNpc neurons are GABAergic and about 1–2% are glutamatergic (Nair-Roberts et al., 2008). It was, therefore, essential to demonstrate that the increased expression of Ca_V_1.3_42_ and Ca_V_1.3_42A_ mRNA resulted from the loss of DA neurons after MPTP treatment and not from changes affecting other neuronal populations. Consistent with this view, the expression of Ca_V_1.3_42_ and Ca_V_1.3_42A_ transcripts remained unchanged in the SNpc when mRNA signals were normalized to either mRNA for GAD1, a GABAergic neuron marker or for Vglut2, a glutamatergic neuron marker.

The upregulation observed in the expression of Ca_V_1.3_42A_ and Ca_V_1.3_42_ mRNA upon normalization to TH and DAT mRNA levels could, therefore, have the following plausible explanations: (i) While the observed change in the expression of Ca_V_1.3 channels in MPTP treated mouse SNpc could be explained as a retention of phenotype, it is important to note that about 50% loss in the number of TH positive neurons along with loss of Nissl positive neurons has been observed in the SNpc of MPTP treated mice (Saeed et al., 2009). Since Ca_V_1.3 channels are expressed in neurons, a resultant reduction in their mRNA expression in SNpc is, therefore, expected concomitant to the reduction in the number of TH positive neurons in SNpc similar to that seen for the mRNA expression of TH and DAT. (ii) The Ca_V_1.3_42A_ and Ca_V_1.3_42_ mRNA upregulation could be a result of their upregulation in non-TH expressing (non-dopaminergic) neurons. To address this issue, we showed that the expression of mRNA for the markers of GABA-ergic neurons, i.e., GAD1 and glutamatergic neurons, i.e., VGlut2 did not change. Further, the expression of Ca_V_1.3_42A_ and Ca_V_1.3_42_ when normalized to GAD1 and VGltu2 expression did not differ in MPTP treated SNpc as compared to controls. (iii) The upregulation of Ca_V_1.3_42A_ and Ca_V_1.3_42_ mRNA results from their increased expression in the surviving TH neurons. In the light of the present results demonstrating a decrease in TH and DAT expression and an increase in Ca_V_1.3_42A_ and Ca_V_1.3_42_ mRNA levels, it is likely that the surviving DA neurons in SNpc are expressing greater levels of Ca_V_1.3_42A_ and Ca_V_1.3_42_ mRNA.

Further, an increase in the expression of Ca_V_1.3_42A_ upon normalization with TH and DAT 24 h after a single exposure to MPTP, in which case, there is no downregulation of TH and DAT also suggests that there is potentially an increase in the expression of Ca_V_1.3 channels in vulnerable DA neurons in SNpc on exposure to MPTP.

This set of results supports the view that mouse DA neurons in SNpc may be more at risk to degenerate presumably because they have to cope with a larger influx of calcium through Ca_V_1.3 L-type calcium channels during neurodegeneration (Surmeier and Schumacker, 2013). Possibly related to present observations, Lieberman et al. (2017) demonstrated that there is a build-up of cytosolic calcium in cultured DA neurons from SN as opposed to VTA following treatment with the active metabolite of MPTP, MPP^+^. To conclude, the sustained high levels of expression of Ca_V_1.3_42_ and its variant Ca_V_1.3_42A_ indicates the possible contribution of these two channel isoforms to degeneration of dopaminergic neurons in the sub-chronic MPTP mouse model of PD.

## Data Availability Statement

The raw data supporting the conclusions of this article will be made available by the authors, without undue reservation, to any qualified researcher.

## Ethics Statement

The animal study was reviewed and approved by Institutional Animal and Ethics Committee of Indian Institute of Science, Bangalore, India (Protocol# CAF/Ethics/267/2012).

## Author Contributions

The research project was conceptualized by VR, organized by AV and VR and executed by AV. Statistical analysis was designed and executed by AV and reviewed and critiqued upon by VR. First draft of the manuscript was written by AV and VR.

## Conflict of Interest

The authors declare that the research was conducted in the absence of any commercial or financial relationships that could be construed as a potential conflict of interest.
