# Supplementary material for: CaV1.3 L-Type Calcium Channels Increase the Vulnerability of Substantia Nigra Dopaminergic Neurons in MPTP Mouse Model of Parkinson’s Disease
Source: Front Aging Neurosci. 2020 Jan 17;11:382. doi: 10.3389/fnagi.2019.00382 (PMC6978652; doi:10.3389/fnagi.2019.00382)
Supplement: Supplementary file 1 [file Data_Sheet_1.pdf]

$Ca_V1.3$ L-type calcium channels increase vulnerability of substantia nigra dopaminergic neurons in MPTP mouse model of Parkinson's disease

Aditi Verma[1] and Vijayalakshmi Ravindranath[1,2*]

[1] Centre for Neuroscience, Indian Institute of Science, Bangalore-560012, India

[2] Centre for Brain Research, Indian Institute of Science, Bangalore-560012, India

***Correspondence:**
Vijayalakshmi Ravindranath,
Centre for Brain Research,
Indian Institute of Science,
C.V. Raman Avenue,
Bangalore - 560012, India,
Phone: +91-80-22933640,
Fax: +91-80-23603323,

email: viji@iisc.ac.in

Keywords: neurodegeneration; SNpc; MPTP; $Ca_V1.3_{42A}$; gene expression; alternative splicing

Supplementary Material

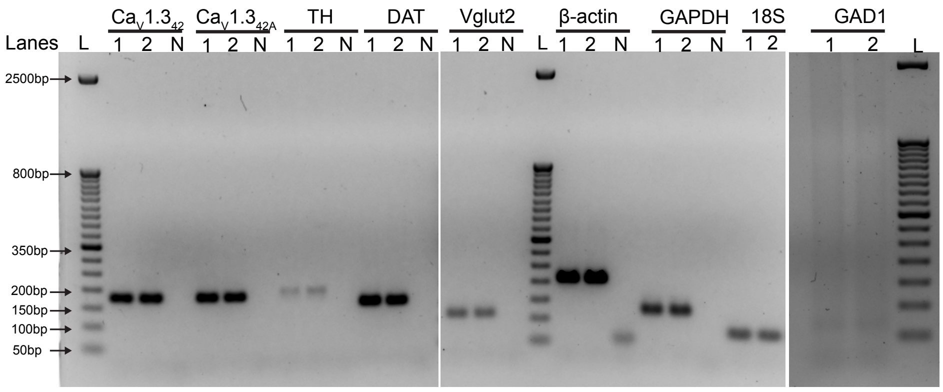

Figure S1: Primer specificity for all the mouse qRT-PCR primers using agarose gel electrophoresis.

Single bands were observed at the desired lengths for the mouse qRT-PCR primers, viz. $Ca_V1.3_{42}$, $Ca_V1.3_{42A}$, TH, DAT, Vglut2, β-actin, GAPDH, 18S and GAD1 using mouse cDNA derived from control tissue. Two SNpc cDNA samples (1 and 2) and a no template negative control (N) was run for each set of qRT-PCR primers. The qRT-PCR products were run on a 2% agarose gel at 100mV. Ladder used was Invitrogen TrackIt™ 50bp DNA ladder (ThermoFisher Scientific).

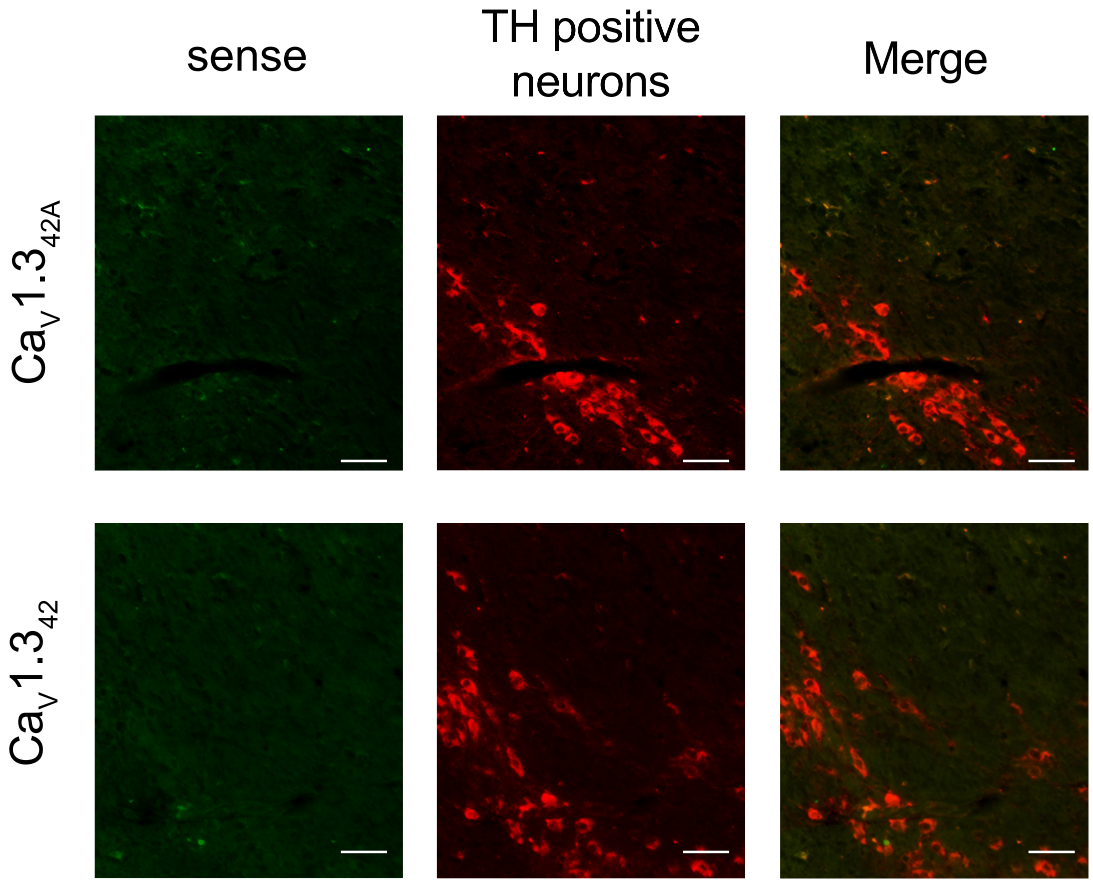

Figure S2: Absence of signal in sections treated with sense *in situ* hybridization probes.

The use of sense probes against $Ca_V1.3_{42A}$ and $Ca_V1.3_{42}$ did not give fluorescent signal within TH positive neurons in the SNpc using fluorescence *in situ* hybridization and TH immunohistochemistry, respectively. Images were acquired using Zeiss Axio imager M2 fluorescence microscope with a 20X/0.8 objective. Scale bar = 50μm.

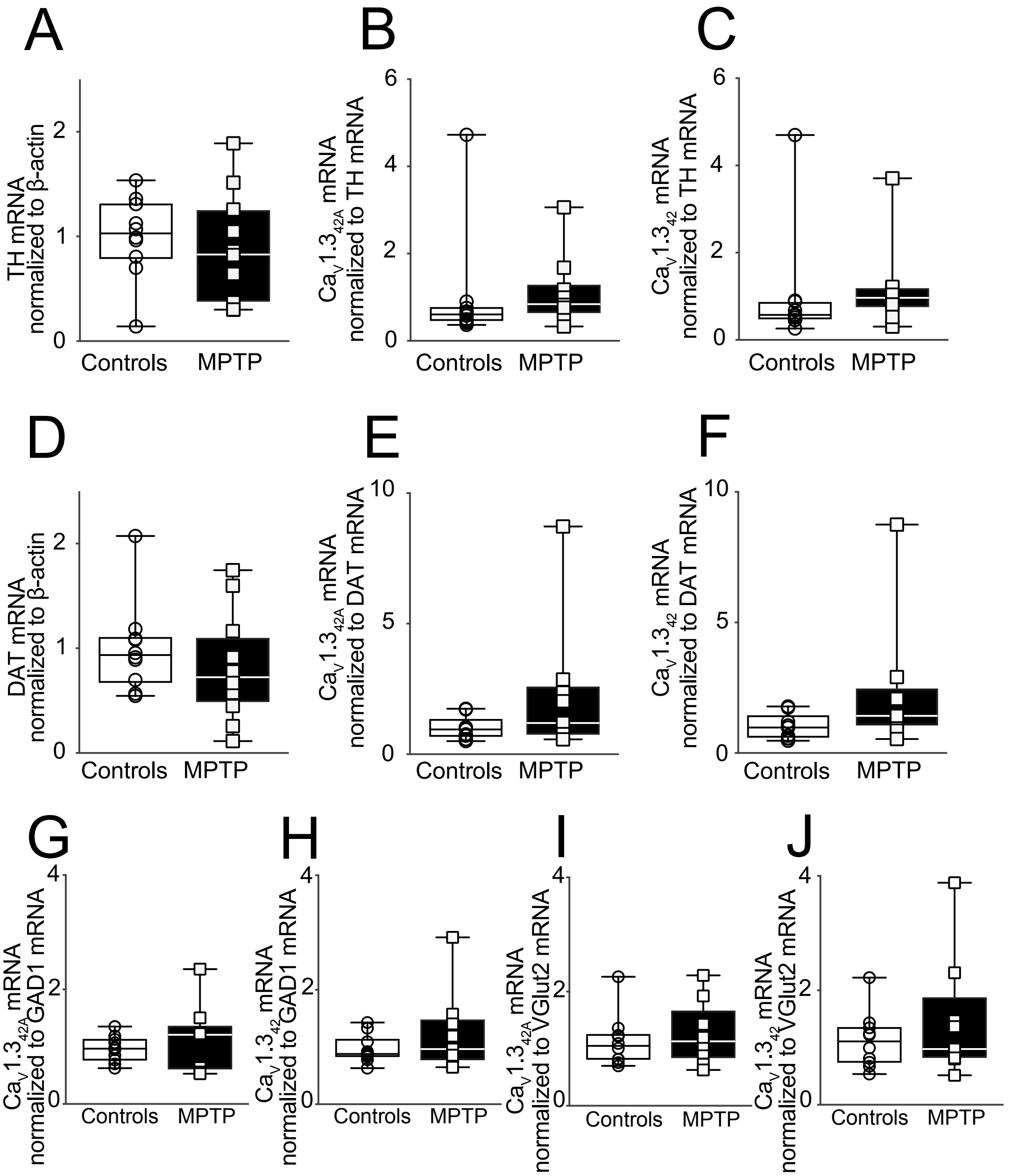

Figure S3: $Ca_V1.3_{42A}$ and $Ca_V1.3_{42}$ mRNA levels in the VTA of MPTP-treated mice.
(A) TH mRNA expression in the VTA of mice treated subcutaneously with MPTP (30mg/kg body weight) for 14 days. MPTP treatment did not lead to significant reduction of TH mRNA levels in the VTA (p=0.6025, t=0.5296, df=19). qRT-PCR data were normalized to mRNA levels of β-actin. **(B)** Relative mRNA levels for $Ca_V1.3_{42A}$ (Mann–Whitney U = 30, n_1 = 10, n_2 = 10, p=0.1431, two-tailed) and **(C)** $Ca_V1.3_{42}$ (Mann–Whitney U = 28, n_1 = 10, n_2 = 10, p=0.1051, two-tailed)were found to be unchanged in the SNpc in response to MPTP treatment when the mRNA signal was normalized to TH. **(D)** mRNA levels of DAT were unchanged upon MPTP treatment when normalized to β-actin mRNA levels (Mann–Whitney U = 45, n_1 = 10, n_2 = 12, p=0.3463, two-tailed). **(E)** Relative mRNA levels for $Ca_V1.3_{42A}$ (Mann–Whitney U = 24, n_1 = 9, n_2 = 9, p=0.1615, two-tailed) and **(F)** $Ca_V1.3_{42}$ (Mann–Whitney U = 22, n_1 = 9, n_2 = 9, p=0.1135, two-tailed) remained unaltered in the VTA in response to MPTP treatment when the mRNA signal was normalized to DAT mRNA levels. **(G)** mRNA levels for

$Ca_V1.3_{42A}$ (Mann–Whitney U = 33, n_1 = 10, n_2 = 9, p=0.3562, two-tailed) and **(H)** $Ca_V1.3_{42}$ (Mann–Whitney U = 35, n_1 = 10, n_2 = 9, p=0.4470, two-tailed) were found to be unchanged in the VTA of MPTP-treated mice upon normalization to GAD1. **(I)** mRNA levels for $Ca_V1.3_{42A}$ (Mann–Whitney U = 40, n_1 = 10, n_2 = 9, p=0.7197, two-tailed)and **(J)** $Ca_V1.3_{42}$ (Mann–Whitney U = 38, n_1 = 10, n_2 = 9, p=0.6038, two-tailed) were unchanged in the VTA of MPTP-treated mice upon normalization to Vglut2. Each point in the scatter represents an individual animal. For controls, n=10, for MPTP treated animals, n=9-10. Unpaired, two-tailed Student's t-test or two-tailed Mann Whitney U was performed for each of the pair-wise comparison. Data represented as box-whiskers plots where each box represents quartiles with the line indicating median. Whiskers show the absolute range.

Table S1: Sequences for mouse qRT-PCR primers

Gene	Primer	Sequence
$Ca_V1.3_{42}$ (NM_028981.2)	Forward primer	GTCCCTCCAGCTGGTGATGATGA
	Reverse primer	GGCCCAATGTCATGCAGGGT
$Ca_V1.3_{42A}$	Forward primer	GTCCCTCCAGCTGGTGATGATGA
	Reverse primer	CAGGCAGAGAACTCTAAAGCATCCG
β -actin (NM_007393.5)	Forward primer	CCTTCTTGGGTATGGAATCCTGTGGC

	Reverse primer	GCGCTCAGGAGGAGCAATGATCTTG
18S rRNA	Forward primer	GAGGGAGCCTGAGAAACGG
	Reverse primer	GTCGGGAGTGGGTAATTTGC
GAPDH (NM_001289726.1)	Forward primer	GGCCTTCCGTGTTCCTAC
	Reverse primer	TGTCATCATACTTGGCAGGTT
TH (NM_009377.1)	Forward primer	GGAACGGTACTGTGGCTACC
	Reverse primer	GAGTGCATAGGTGAGGAGGC
DAT (NM_010020.3)	Forward primer	GGTTCTACGGTGTCCAGCAA

	Reverse primer	TAGTGTGGGGGTCTGAAGGT
Vglut2(NM_080853.3)	Forward primer	GCGGAGGCAAAGTTATCAAG
	Reverse primer	CCTGGAATCTGGGTGATGAT
GAD1(NM_008077.5)	Forward primer	GGGTGGTGGACTGCTCATGT
	Reverse primer	AATTGGCCCTTTCTATGCCG

Table S2: qRT-PCR conditions

PCR step	Temperature	Time	Cycles
Initial Denaturation	95^0C	10min	1
Denaturation	95^0C	20s	40
Annealing	60^0C	30s	40

Elongation	72^0C	40s	40
