# Supplementary material for: CaV1.3 L-Type Calcium Channels Increase the Vulnerability of Substantia Nigra Dopaminergic Neurons in MPTP Mouse Model of Parkinson’s Disease
Source: Front Aging Neurosci. 2020 Jan 17;11:382. doi: 10.3389/fnagi.2019.00382 (PMC6978652; doi:10.3389/fnagi.2019.00382)
Supplement: Supplementary file 2 [file Table_1.DOCX]

Ca_V_1.3 L-type calcium channels increase vulnerability of substantia nigra dopaminergic neurons in MPTP mouse model of Parkinson’s disease

Aditi Verma^1^ and Vijayalakshmi Ravindranath^1,2*^

^1^ Centre for Neuroscience, Indian Institute of Science, Bangalore-560012, India

^2^ Centre for Brain Research, Indian Institute of Science, Bangalore-560012, India

***Correspondence:**Vijayalakshmi Ravindranath,

Centre for Brain Research,

Indian Institute of Science,

C.V. Raman Avenue,

Bangalore - 560012, India,

Phone: +91-80-22933640,

Fax: +91-80-23603323,

email: viji@iisc.ac.in

Keywords: neurodegeneration; SNpc; MPTP; Ca_V_1.3_42A_; gene expression; alternative splicing

Supplementary Material

**Figure S1: Primer specificity for all the mouse qRT-PCR primers using agarose gel electrophoresis.**

Single bands were observed at the desired lengths for the mouse qRT-PCR primers, viz. Ca_V_1.3_42_, Ca_V_1.3_42A_, TH, DAT, Vglut2, β-actin, GAPDH, 18S and GAD1 using mouse cDNA derived from control tissue. Two SNpc cDNA samples (1 and 2) and a no template negative control (N) was run for each set of qRT-PCR primers. The qRT-PCR products were run on a 2% agarose gel at 100mV. Ladder used was Invitrogen TrackIt™ 50bp DNA ladder (ThermoFisher Scientific).

**Figure S2: Absence of signal in sections treated with sense *in situ* hybridization probes.**

The use of sense probes against Ca_V_1.3_42A_ and Ca_V_1.3_42_ did not give fluorescent signal within TH positive neurons in the SNpc using fluorescence *in situ* hybridization and TH immunohistochemistry, respectively. Images were acquired using Zeiss Axio imager M2 fluorescence microscope with a 20X/0.8 objective. Scale bar = 50μm.

**Figure S3:** **Ca_V_1.3_42A_ and Ca_V_1.3_42_ mRNA levels in the VTA of MPTP-treated mice.**

(A) TH mRNA expression in the VTA of mice treated subcutaneously with MPTP (30mg/kg body weight) for 14 days. MPTP treatment did not lead to significant reduction of TH mRNA levels in the VTA (p=0.6025, t=0.5296, df=19). qRT-PCR data were normalized to mRNA levels of β-actin. (B) Relative mRNA levels for Ca_V_1.3_42A_ (Mann–Whitney U = 30, n_1_ = 10, n_2_ = 10, p=0.1431, two-tailed) and (C) Ca_V_1.3_42_ (Mann–Whitney U = 28, n_1_ = 10, n_2_ = 10, p=0.1051, two-tailed)were found to be unchanged in the SNpc in response to MPTP treatment when the mRNA signal was normalized to TH. (D) mRNA levels of DAT were unchanged upon MPTP treatment when normalized to β-actin mRNA levels (Mann–Whitney U = 45, n_1_ = 10, n_2_ = 12, p=0.3463, two-tailed). (E) Relative mRNA levels for Ca_V_1.3_42A_ (Mann–Whitney U = 24, n_1_ = 9, n_2_ = 9, p=0.1615, two-tailed) and (F) Ca_V_1.3_42_ (Mann–Whitney U = 22, n_1_ = 9, n_2_ = 9, p=0.1135, two-tailed) remained unaltered in the VTA in response to MPTP treatment when the mRNA signal was normalized to DAT mRNA levels. (G) mRNA levels for Ca_V_1.3_42A_ (p=0.329, t=1.007, df=17) (Mann–Whitney U = 33, n_1_ = 10, n_2_ = 9, p=0.3562, two-tailed) and (H) Ca_V_1.3_42_ (Mann–Whitney U = 35, n_1_ = 10, n_2_ = 9, p=0.4470, two-tailed) were found to be unchanged in the VTA of MPTP-treated mice upon normalization to GAD1. (I) mRNA levels for Ca_V_1.3_42A_ (Mann–Whitney U = 40, n_1_ = 10, n_2_ = 9, p=0.7197, two-tailed)and (J) Ca_V_1.3_42_ (Mann–Whitney U = 38, n_1_ = 10, n_2_ = 9, p=0.6038, two-tailed) were unchanged in the VTA of MPTP-treated mice upon normalization to Vglut2. Each point in the scatter represents an individual animal. For controls, n=10, for MPTP treated animals, n=9-10. Unpaired, two-tailed Student’s t-test or two-tailed Mann Whitney U was performed for each of the pair-wise comparison. Data represented as box-whiskers plots where each box represents quartiles with the line indicating median. Whiskers show the absolute range. *p<0.05.

Supplementary tables of qRT-PCR primer sequences:

**Table S1: Sequences for mouse qRT-PCR primers**

| Gene | Primer | Sequence |
| --- | --- | --- |
| Ca_V_1.3_42_  (NM_028981.2) | Forward primer | GTCCCTCCAGCTGGTGATGATGA |
|  | Reverse primer | GGCCCAATGTCATGCAGGGT |
| Ca_V_1.3_42A_ | Forward primer | GTCCCTCCAGCTGGTGATGATGA |
|  | Reverse primer | CAGGCAGAGAACTCTAAAGCATCCG |
| β -actin (NM_007393.5) | Forward primer | CCTTCTTGGGTATGGAATCCTGTGGC |
|  | Reverse primer | GCGCTCAGGAGGAGCAATGATCTTG |
| 18S rRNA | Forward primer | GAGGGAGCCTGAGAAACGG |
|  | Reverse primer | GTCGGGAGTGGGTAATTTGC |
| GAPDH (NM_001289726.1) | Forward primer | GGCCTTCCGTGTTCCTAC |
|  | Reverse primer | TGTCATCATACTTGGCAGGTT |
| TH (NM_009377.1) | Forward primer | GGAACGGTACTGTGGCTACC |
|  | Reverse primer | GAGTGCATAGGTGAGGAGGC |
| DAT (NM_010020.3) | Forward primer | GGTTCTACGGTGTCCAGCAA |
|  | Reverse primer | TAGTGTGGGGGTCTGAAGGT |
| Vglut2(NM_080853.3) | Forward primer | GCGGAGGCAAAGTTATCAAG |
|  | Reverse primer | CCTGGAATCTGGGTGATGAT |
| GAD1(NM_008077.5) | Forward primer | GGGTGGTGGACTGCTCATGT |
|  | Reverse primer | AATTGGCCCTTTCTATGCCG |

**Table S2: qRT-PCR conditions**

| PCR step | Temperature | Time | Cycles |
| --- | --- | --- | --- |
| Initial Denaturation | 95^0^C | 10min | 1 |
| Denaturation | 95^0^C | 20s | 40 |
| Annealing | 60^0^C | 30s | 40 |
| Elongation | 72^0^C | 40s | 40 |
